# The Injectable Woven Bone-Like Hydrogel to Perform Alveolar Ridge Preservation With Adapted Remodeling Performance After Tooth Extraction

**DOI:** 10.3389/fbioe.2020.00119

**Published:** 2020-02-21

**Authors:** Tao Yang, Peng Xie, Zhenzhen Wu, Yunmao Liao, Wenchuan Chen, Zhichao Hao, Yushu Wang, Zhimin Zhu, Wei Teng

**Affiliations:** ^1^Department of Prosthodontics, Guanghua School of Stomatology, Hospital of Stomatology, Guangdong Provincial Key Laboratory of Stomatology, Sun Yat-sen University, Guangzhou, China; ^2^State Key Laboratory of Oral Diseases, National Clinical Research Center for Oral Diseases, West China Hospital of Stomatology, Sichuan University, Chengdu, China; ^3^Department of Periodontology and Implantology, Stomatological Hospital, Southern Medical University, Guangzhou, China; ^4^Department of Biomedical Engineering, Tufts University, Medford, MA, United States

**Keywords:** alveolar ridge preservation, woven bone, injectable hydrogel, remodeling, biomimetic, amorphous calcium phosphate, mineralized collagen fibril

## Abstract

Grafting bone substitute is paramount to prevent the alveolar ridge resorption after tooth extraction and facilitate the subsequent implant treatment. An ideal bone substitute should acquire the excellent osteogenic property, more importantly, possess the suitable remodeling rate in balance with bone formation and desirable clinical manageability. However, none of bone substitute is simultaneously characterized by these features, and currently, the limited remodeling property leads to the excessive waiting time before implantation. Enlightened by woven bone, the transitional tissue that is able to induce osteogenesis during bone healing could be easily remodeled within a short period and depend on the favorable injectability of hydrogel, an injectable woven bone-like hydrogel (IWBLH) was constructed in this study to address the above problems. To mimic the component and hierarchical structure of woven bone, amorphous calcium phosphate (ACP) and mineralized collagen fibril were synthesized and compounded with alginate to form IWBLHs with various ratio. Screened by physiochemical characterization and *in vitro* biological assays, an optimal IWBLH was selected and further explored in rat model of tooth extraction. Compared with the most widely used bone substitute, we showed that IWBLH could be easily handled to fully fill the tooth socket, perform a comparable function to prevent the alveolar bone resorption, and completely remodeled within 4 weeks. This IWBLH stands as a promising candidate for alveolar ridge preservation (ARP) in future.

## Introduction

The dental implantation is currently representing the mainstream option for replacing the missing teeth. Sufficient alveolar bone is the fundamental prerequisite for the implant survival and success. Unfortunately, the alveolar bone is subjected to a huge resorption after tooth extraction, characterized by a 40–60% bone loss in the height and width of the residual alveolar ridge (Farmer and Darby, 2014). The severe alveolar bone loss could limit the feasibility of implantation and complicate the restoration procedure (MacBeth et al., 2017). To address this issue, the alveolar ridge preservation (ARP) techniques have been proposed. Among various ARP techniques, filling the tooth socket with the bone substitute following the tooth extraction is a proven and effective approach (Kalsi et al., 2019).

The rationale of bone substitute is to promote the tooth socket healing process (Komlev et al., 2015), thereby reducing the residual alveolar ridge resorption. The ideal bone substitute should highly induce host cells to assume specialized functions during bone formation. Establishing the biomimetic bone extracellular matrix (ECM) mimicking the bone microenvironment has been evidenced to be a straightforward strategy to accelerate the bone healing (Ding et al., 2019). Meanwhile, the ideal bone substitute should be gradually remodeled into vital bone and the remodeling rate should closely match to the rate of bone formation (Subramaniam et al., 2016). The premature remodeling often results in the incomplete bone healing and impairs ARP effect. The delayed remodeling could disturb the early stage of the extraction socket healing (Zhou et al., 2017), which significantly postpones the time of implant placement (at least 8 months delay) and severely prolongs the overall treatment time. Although plenty of bone substitutes have been widely used in the clinic, the majority of them are characterized by a slow remodeling nature. Therefore, constructing a biomimetic bone substitute with an adapted remodeling rate is a pivotal challenge need to be addressed.

Woven bone, an emerging immature bone during tooth socket healing (Shah et al., 2019), behaves as an optimally temporary scaffold for the deposition of cells involved in bone formation and regulating their bioactivities (Paino et al., 2017). Previous studies have demonstrated that grafting the fracture callus rich in woven bone was a promising approach treating the non-union (Zhang et al., 2010). Through the activities of osteoblasts and osteoclasts, mature bone lays down on the surface of woven bone and woven bone was completely remodeled within a short period (Schropp et al., 2003). Of note, the remodeling of woven bone is somewhat synchronized with the formation of mature bone. Theoretically, engineering a biomimetic woven bone would have tremendous potential to simultaneously accelerate the tooth socket healing and solve the remodeling problem encountered in current bone substitute.

The hierarchical structure and component are the critical factors when simulating woven bone in order to obtain relevant biological behavior. For the hierarchical structure, woven bone, featured by a low mineral content, is considered as an organic matrix scaffold where coarse mineralized collagen fibrils are haphazardly distributed (Su et al., 2003; Li Y. et al., 2011). The organic matrix scaffold serves as a 3D framework for cell growth (Lohmann et al., 2017; Feng et al., 2019). The mineralized collagen fibrils, collagen fibrils with nano-hydroxyapatite (nHA) deposited both intrafibrillarly and extrafibrillarly, exert vital functions to regulate osteoblastic differentiation of bone marrow mesenchymal stem cells (BMMSCs) and promote the bone formation (Liu et al., 2016a; Lausch et al., 2018). As for the component, besides collagen and nHA, abundant amorphous calcium phosphate (ACP) could be detected in woven bone. ACP is a precursor phase of bone mineral and is extremely rich during the bone formation (Combes and Rey, 2010). Its unique properties especially for the capacity of sustained release of calcium ion could also generate multi-biofunction during osteogenesis (Tadic et al., 2002). Hence, the core factors for mimicking woven bone lie in constructing organic matrix scaffold, mineralized collagen fibrils, and ACP.

Clinical manageability is the other key issue designing the biomimetic woven bone employed in APR. As the geometries of tooth socket are usually complicated, it is quite difficult to prefabricate graft that exactly fits the irregularly shaped socket. Meanwhile, the current granular shaped graft is incapable to steadily maintain inside the socket. The injectable hydrogel is currently gaining increasing interests for its self-setting property (Choi et al., 2018; Wang G. et al., 2018). After injected, the sol could perfectly fill the irregular socket and become a scaffold maintaining inside socket when the gel forms *in situ*. Alginate is naturally derived polysaccharide with excellent biocompatibility and shares similarity to the ECM (Kahl et al., 2019). Its abundant carboxylic acid could not only promote the osteogenic differentiation (Venkatesan et al., 2015) and but also be cross-linked to form scaffold in the presence of calcium ion (Turco et al., 2009), which make alginate a potent candidate in constructing biomimetic woven bone. However, directly introducing the external calcium ion results in immediately forming of inhomogeneous scaffold and the extremely short sol–gel transition time could not satisfy the clinical practice. Interestingly, ACP could sustainably release calcium ion which, hypothetically, could effectively solve the overquick gelation problem of alginate.

**FIGURE 1:**
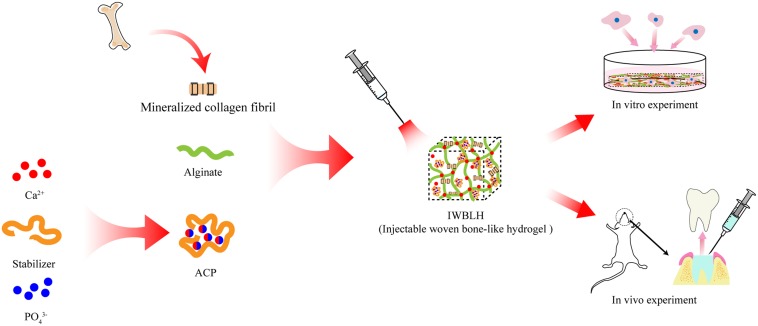
The scheme illustrating the construction process of IWBLH.

The present studies described our attempt to develop an injectable woven bone-like hydrogel (dominated as IWBLH), which is composed of mineralized collagen fibrils, ACP, and alginate, to mimic the hierarchical structure and component of woven bone with satisfying manageability in ARP technique. Cross-linked by the calcium ion released from ACP, the injectable alginate is supposed to form the scaffold, where the coarse mineralized collagen fibril was randomly distributed. The physiochemical property of IWBLH and the effect on BMSCs behavior were assessed; its ARP function and remodeling performance *in vivo* were further explored *via* the tooth extraction model of rat (Figure 1). Through this study, a novel injectable bone substitute with excellent osteogenesis performance and suitable remodeling rate is supposed to be exploited in order to achieve better ARP effect.

## Materials and Methods

### The Synthesis of ACP

The ACP was synthesized according to the previous method (Yang et al., 2016). In brief, 50 mg carboxymethyl chitosan (CMCS) (Xiya Reagent Co., Ltd., Chengdu, China) was dispersed in Tris-HCl buffer containing 100 mg CaCl_2_ and resulting CMCS-Ca solution was agitated overnight and pH was kept at 8.0, followed by dropwise adding 137.1 mg of K_2_HPO_4_ solution. The pH of resulting ACP solution was maintained at 8.0. After 30 min agitation, the ACP solution was filtrated, washed by water and ethanol, and freeze-dried. The product was characterized by Fourier transform infrared (FTIR) spectroscopy (Nicolet 6700, Thermo Nicolet Corp., Madison, WI, United States), powder X-ray diffractometry (XRD) (Bruker D8 Advance, Bruker, Germany), transmission electron microscopy (TEM) (FEI, Hillsboro, OR, United States), selected area electron diffraction (SAED), and scanning electron microscopy (SEM) (Inspect F50, FEI, Hillsboro, OR, United States). The phase transformation of ACP was evaluated by immersing ACP into the PBS for 24 h, after which the microstructure and the crystalline were characterized. The bone integrity ability of ACP was tested in the following method. A bone block, half of which was sealed by the 3 M tape, was immersed in the fresh prepared ACP dispersion for the 24 h. After that, the bone was taken out and vigorously shaken in water at 100 r/min for 1 h to remove the loosely attached ACP. Then, the sample was dried and observed under the SEM.

### The Synthesis of Alginate Hydrogel Cross-Linked by ACP

2 wt% Sodium alginate (SA) solution was prepared by dissolving 100 mg SA (Kelong Reagent Co., Ltd., Chengdu, China) in PBS. Then, different amounts of lyophilized ACP were well-dispersed in PBS and added to the agitating SA solution drop by drop at 37°C with the SA/ACP weight ratios of 2:2, 2:3, 2:4, 2:5, and 2:6, respectively. The sol–gel transition time was determined by the test tube inverting method (Hsiao et al., 2012). Briefly, 10 ml SA/ACP sol sample was added into 15 ml tube placed in the 37°C water bath. The tube was taken out and inverted at the designed time intervals to test the gelation behavior. No flow within 30 s was set as the criterion for gel state. The as-formed SA/ACP hydrogel was lyophilized and further subjected to SEM, XRD, FTIR, and thermogravimetric analysis (TGA) (STA 449/C Jupiter, Netzsch, Selb, 137 Germany). The phase transformation of SA/ACP hydrogel was also evaluated *via* XRD and SEM after immersing SA/ACP hydrogel into the PBS for 24 h.

### The Preparation of Antigen-Extracted Mineralized Collagen Fibril

The cancellous bones of pig vertebrae were cut into cuboids with dimension around 5 mm × 5 mm × 3 mm and cleaned by the distilled water. To remove the antigen (Li D. et al., 2011), the cuboids were vigorously agitated in the mixture of methanol and chloroform (v/v was 1:1) for 24 h and immersed in 30% hydrogen peroxide for 12 h. Then, the cuboids were further subjected to the stirring in mixture of methanol and chloroform for another 24 h. The antigen-extracted cuboids were fully rinsed with distilled water and kept in freezer overnight. Subsequently, the bone cuboids were ground in the planetary mill (QXQM-4L, Changsha Tianchuang Powder Technology Co. Ltd., China) for 2 h with the speed of 400 r/min. During the grinding, the temperature was kept below 45°C by adding the ice into the milling jar every 30 min. The dispersion of mineralized collagen fibril was sieved (400 mesh), collected, freeze-dried, sterilized by Co 60 radiation, and further characterized by SEM, EDS, HRTEM, SAED, XRD, and TGA. The cytotoxicity was conducted *via* testing the extracted medium of mineralized collagen fibril as described in Section “*In vitro* Cytotoxicity Assay of IWBLHs.”

### The Preparation of IWBLH

A series of IWBLHs were prepared by dispersing different doses of mineralized collagen fibril into 2 wt% SA solution *via* agitation, followed by adding different dose of ACP dispersion solution. Table 1 summarizes the detailed doses of SA, ACP, and mineralized collagen fibril in each IWBLH group. The sol–gel transition time was tested by the method mentioned in Section “The Synthesis of Alginate Hydrogel Cross-Linked by ACP” and rheometer (Anton Paare Physica MCR 302, Germany). The resulting gel was freeze-dried and characterized by SEM and TGA. The porosity IWBLH was evaluated by the previously reported method (Jiang et al., 2008). The weight (*W*_0_) and volume (*V*_0_) of lyophilized IWBLH were measured. Then, the IWBLH was immersed in ethanol and its weight (*W*_1_) was recorded until it was saturated by ethanol. The porosity was calculated according to the following equation: *The porosity (%)* = *(W_1_−W_0_)* × *100%/(0.789* × *V_0_).* The *in vitro* degradation of IWBLH was performed following the previous published protocol (Liu et al., 2014). A series of freeze-dried IWBLHs were immersed in PBS at 37°C for up to 28 days. The PBS was refreshed every other day. At the predetermined time, the IWBLH was dehydrated by ethanol and dried in an oven at 37°C. The weight loss percentage was expressed as the ratio of remaining weight and initial weight.

**TABLE 1:** Doze of SA, mineralized collagen fibril, and ACP in IWBLH (unit: mg).

**IWBLH**	**Weight ratio of SA/ACP/mineralized collagen fibril**	**SA**	**ACP**	**Mineralized collagen fibril**
Group A	2:3:2	100	150	100
Group B	2:4:2	100	200	100
Group C	2:4:3	100	200	150
Group D	2:5:2	100	250	100
Group E	2:5:3	100	250	150
Group F	2:5:4	100	250	200

### Cell Culture

Human osteoblast-like cells MG63 cell line was purchased from ATCC company. The cells were culture in DMEM supplemented with 10% FBS and 1% penicillin/streptomycin at 37°C with 5% CO_2_ and passage number 10-15 was used. Bone marrow stromal cells (BMSCs) were extracted from the femoral marrow of 3-week female Sprague–Dawley (SD) rats. The cells were cultured in α-MEM with 10% FBS and 1% of penicillin/streptomycin. BMSCs from passage 3 were used for further experiments.

### *In vitro* Cytotoxicity Assay of IWBLHs

The cytotoxicity of hydrogel was evaluated by CCK8 assay (Dojindo, Japan). The extracted medium was prepared by immersing the sterilized IWBLHs in DMEM culture media supplemented with 10% FBS and 1% penicillin/streptomycin at 37°C for 24 h. The extraction ratio of IWBLH to culture media was 0.2 g/ml. MG63 cell was seeded in 96-well plate at a seeding density of 5000 per well. After 24 h, the 150 μl of different concentration of extracted media filtered through 0.22 μm filter was added to each well and the cells were further incubated for 24 or 48 h. Then, the media were replaced by fresh media containing 20 μl CCK8. 3 h later, the absorbance was measured at 450 nm. Cells treated with culture media were considered as controls and its absorbance was used to normalize the cytotoxicity of extract. Triplicate samples from each group were used to evaluate the cytotoxicity.

### The Cell Attachment Assay

To test the cell attachment property of the IWBLHs, the cell attachment assay was conducted according to the previous paper with minor modification (Thein-Han and Misra, 2009; Budiraharjo et al., 2012). The sterilized freeze-dried IWBLHs were placed in 24-well plate. The IWBLHs were saturated in the culture media and extra media was removed; 2 × 10^4^ BMSCs were seeded to each IWBLH and incubated for 6 h. Then, the IWBLH was carefully rinsed with culture media prior to being taken out in order to remove weakly attached cells. Then, the BMSCs remained in 24-well plate belonged to the unattached cells, which were trypsinized and the number was calculated by automated cell counter. The percentage of attached cell could be calculated *via* the following equation: *The attachment percentage* = *1 − unattached cell number/initial seeding cell number*.

### The Effect of IWBLHs on Proliferation and Osteogenic Differentiation of BMSCs

The IWBLHs were placed in 48-well plate and prewetted with BMSC culture media; 5 × 10^3^ BMSCs were seeded to each IWBLH. The media was changed every 2 days. At the 7, 14, and 21 days, the proliferation of BMCSs was tested *via* CCK8 assay. For osteogenic differentiation assay, 2 × 10^4^ BMSCs were seeded to IWBLH in 48-well plate. One day later, the media was changed to the osteogenic medium (BMSC culture media supplemented with 50 μM ascorbic acid, 10 μM dexamethasone, and 10 mM β-glycerophosphate). The media was changed every 2 days. At the predetermined time, the alkaline phosphatase (ALP) and osteocalcin (OCN) of cells were tested using the ALP assay kit (Beyotime Institute of Biotechnology, China) and OCN ELISA kit (Nanjing Jiancheng Bioengineering Institute, China), respectively. Based on the above characterization of physicochemical properties and bioactivities, the optimal IWBLH was selected and employed in subsequent *in vivo* study.

### The Rat Model of Tooth Extraction

The tooth extraction of rat model was established based on the previously reported method (Willett et al., 2017). Briefly, 48 SD rat (6-week, female) were acclimated for 1 week and randomly divided into two groups prior to the surgery. Group A was injected with IWBLH and group B was grafted with bone xenograft (Bio-Oss, Geistlich, Switzerland). All animal care and experiments were conducted under the supervision of the Animal Research Committee of the West China School of Stomatology, Sichuan University (WCHSIRB-D-2018-144). The rats were administered general anesthesia through intraperitoneal injection of ketamine (100 mg/kg, Yuhan, South Korea). The mouth was opened *via* the mouth gag and maxillary first molar (M1) of both sides were extracted in a minimally invasive way. Then, the standardized tooth extraction socket was created by removing the interradicular septum of M1. Using a #4 round dental bur, the bone of interradicular septum was removed until the depth from socket bottom to cementoenamel junction of second molar (M2) reached 2 mm. Either IWBLH or bone xenograft was grafted into one side of tooth socket, the other side was unmanipulated and served as the blank control. Subsequently, the tooth sockets were covered with collagen membrane (Bio-Gide, Geistlich, Switzerland) and the mucosa was sutured. The wound healing was carefully monitored after surgery.

### Micro CT Evaluation

Six rats in each group were sacrificed at the predetermined time (1, 2, 3, and 4 weeks) and the maxillary bones were collected and immersed in 10% neutral buffered formalin for 48 h prior to the radiographic measurement. The fixed maxillae were scanned by micro CT measurement (lCT 80; Scanco Medical, Bassersdorf, Switzerland). The settings were as followed: voltage 70 kV, electric current 200 μA, exposure time of 300 ms. The images were reconstructed with an isotropic voxel size of 10 μm. The measurement of height, width, and bone density of alveolar ridge was conducted according to the previous method with minor modification (Willett et al., 2017). Briefly, the coronal slices were employed to evaluate the buccal and palatal height of alveolar ridge as well as the width. The coronal image, which could capture the best anatomy of mesial canals of the M2 and align with the line best fitting through the palatal and buccal cementoenamel junction of M2, was set as the reference plane. Parallel to this plane, the height and width in the mid-socket slice of M1 were measured. The sagittal image was used to measure mesial and distal height of alveolar ridge. The reference views were set to best present the mesial and distal canals of M2, where the mesial and distal height of M1 ridge was measured. In addition, the bone density was analyzed in the middle area of M1 tooth socket, where the region of interest was set as 0.5 mm × 0.5 mm × 0.5 mm and the bone density was measured.

### Histological Evaluation

Following measurement by micro CT, the samples were dehydrated in an ascending graded series of ethanol (20, 40, 60, 80, 90, and 100%), embedded in paraffin, sectioned, and stained with hematoxylin–eosin (HE) and Masson trichrome trichrome. The socket healing and bone substitute remodeling status were assessed by light microscopy (BX51, Olympus, Japan).

### Statistical Analysis

Statistical analyses were performed with SPSS-PC 17.0 Software (SPSS Inc., Chicago, IL, United States). The data were described as the mean ± standard deviation. The one-way ANOVA and LSD tests were used to determine statistical significance. The level of significance was set as *p* < 0.05.

## Results

### The Synthesis of ACP and SA/ACP Hydrogel

Featured by an irregular shape (shown in Supplementary Figure S1), the synthesized ACP possessed an amorphous hump (about 30°) in XRD spectrum and a single peak of PO_4_^3–^ bending mode (around 552 cm^–1^) in FTIR spectrum, indicating that this calcium phosphate was structurally non-crystalline. After 24 h immersion in PBS, the characteristic peak of HA (around 25.8° and 31.8°) was detected in XRD spectrum, two split peaks (530 and 565 cm^–1^) was observed in FTIR spectrum, and nanoneedle-like structure was found in SEM and TEM image, implying that ACP was conversed to nHA. In addition, the ACP exhibited a strong integrity capacity to the bone tissue. Shown in Supplementary Figure S1D, a layer of substance firmly attaching to the bone was formed on the bone surface exposed to the ACP dispersion. EDS showed that it was composed of Ca and P, indicating that this attached layer was generated from ACP.

**FIGURE 2:**
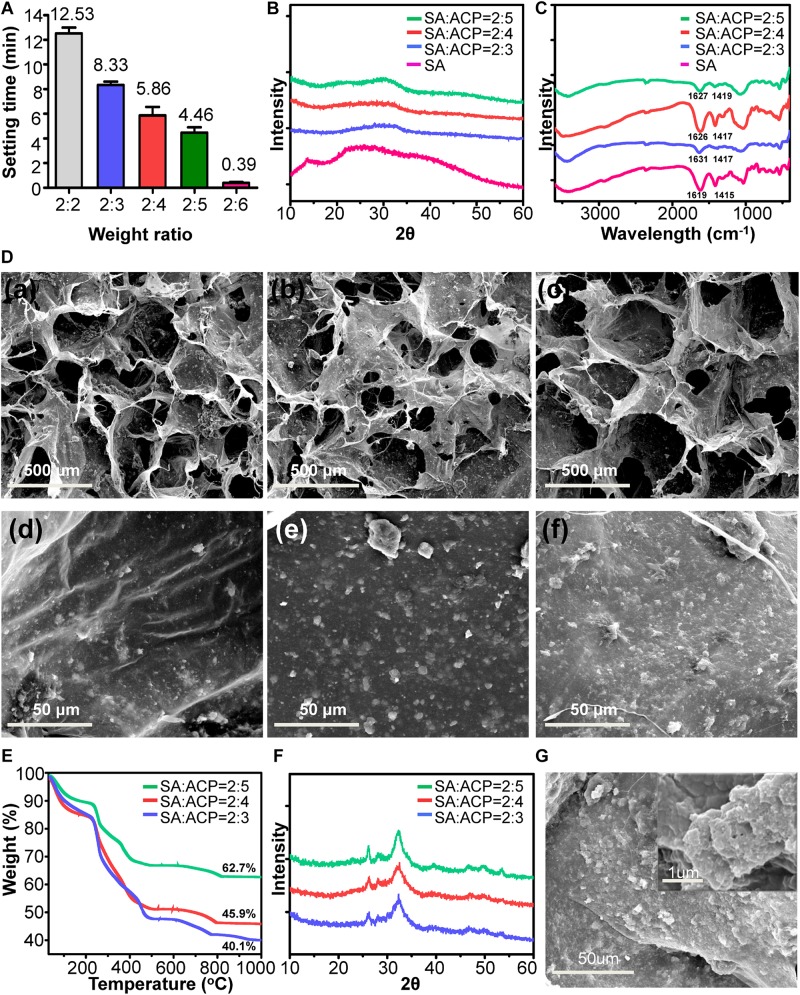
The physiochemical characterization of SA/ACP hydrogels. **(A)** The sol–gel transition times of hydrogels with different weight ratio, the weight ratio indicated SA: ACP (*n* = 5, experiments performed in triplicate. The bars show the mean ± SD). **(B)** XRD and **(C)** FTIR spectra of fresh prepared SA/ACP hydrogels. **(D)** Representative SEM images of hydrogels. **(a,d)**, **(b,e)**, and **(c,f)** represented SA/ACP hydrogels with weight ratio of SA to ACP was 2:3, 2:4, and 2:5, respectively. **(E)** TGA results for SA/ACP hydrogels. **(F)** XRD spectra of SA/ACP hydrogels immersed in PBS for 24 h. **(G)** Representative SEM image of SA/ACP hydrogel immersed in PBS for 24 h.

The sol–gel transition times of series of SA/ACP hydrogel were tested *via* the tube inverting method. As shown in Figure 2A, the time gradually shortened with the decrease of the weight ratio of SA to ACP from 2:2 to 2:6. According to the previously reported data (Van den Vreken et al., 2010) and the clinical experience, the recommended setting time range was set from 2 to 11 min. Therefore, the groups with weight ratios of 2:2 and 2:6 were excluded because of the excessively slow or fast gelation process. As shown in Figure 2B, with the addition of ACP, the crystallinity peak of SA (13.6°) disappeared, indicating that the ACP was uniformly distributed in the SA/ACP hydrogel. Meanwhile, the amorphous diffraction peak (around 30°) was detected in the XRD spectra of hydrogel, reflecting that ACP was not dramatically altered during the gelation process. The FTIR results (Figure 2C) showcased that the peaks of symmetric carboxyl (1415 cm^–1^) and asymmetric carboxyl (1619 cm^–1^) bands of SA shifted to higher wavenumbers in SA/ACP hydrogel, indicating the chelating interaction between of calcium and carboxyl group of SA. The SEM (Figure 2D) exhibited that three hydrogels were all featured by the porous structures with good interconnection. The pore sizes were approximately 400–600 μm. As presented in the magnified image, the ACP was evenly distributed in the pore wall. With the weight ratio of ACP increased, the surface roughness of pore wall gradually enhanced and the weight percentage of inorganics increased (Figure 2E). Additionally, similar to the phase transformation process of ACP, the ACP embedded in SA/ACP hydrogel could transform into nHA after 24 h in PBS (Figures 2F,G), indicated that the physical behavior of ACP was not affected by SA.

### The Characterization of Antigen-Extracted Mineralized Collagen Fibril

**FIGURE 3:**
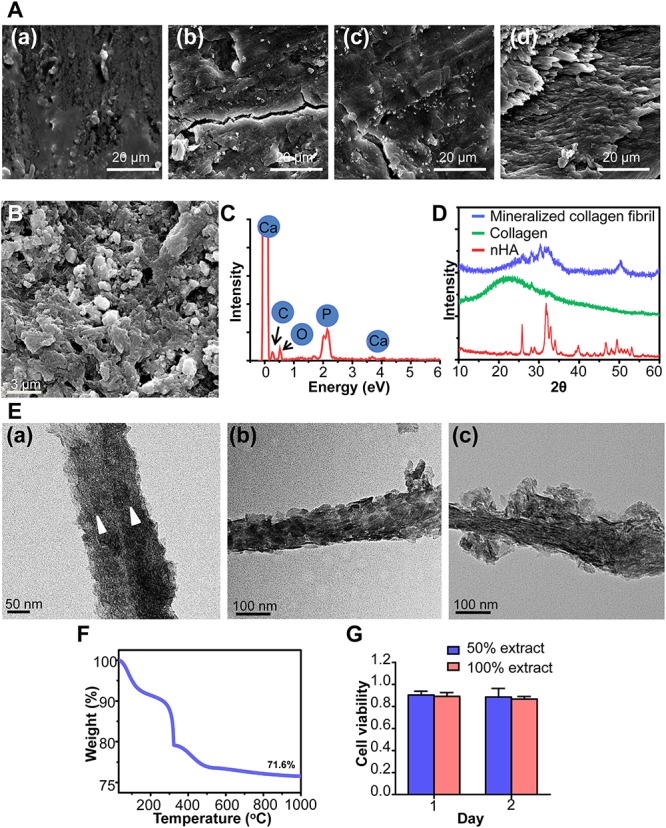
The characterization of antigen-extracted mineralized collagen fibril. **(A)** The SEM image of bone during the process. **(a)** Fresh bone, bone subjected to the first methanol and chloroform process **(b)**, hydrogen peroxide process **(c)**, and second methanol and chloroform process **(d)**. **(B)** The SEM and **(C)** EDX spectrum of mineralized collagen fibril. **(D)** The XRD spectra. **(E)** Representative TEM images of mineralized collagen fibril. The triangles in **(a)** indicated the individual mineralized collagen fibrils merged into coarse bundle, the face-on **(b)** and edge-on **(c)** view of nHA in mineralized collagen fibril. **(F)** The TGA result of mineralized collagen fibril following combusted to 1000°C in the air. **(G)** The different concentrations of extract of mineralized collagen fibril on the viability of MG63 cell (*n* = 4, experiments performed in triplicate. The bars show the mean ± SD).

Through a series of process, the sludge-like fat and protein in bone has been efficiently removed (Figure 3A), which was confirmed by TGA result (Supplementary Figure S2A). The crystalline of nHA was not significantly destroyed (Supplementary Figure S2B). After milling, the resulting antigen-extracted mineralized collagen fibril exhibited a thread-like morphology (Figure 3B), which contained calcium and phosphate (Figure 3C). The typical peaks for HA (25.8° and 31.8°) could be observed in the XRD (Figure 3D), indicating that the inorganics was not significantly destroyed during the process. The broad peak around 25.8° suggested that nHA was featured by the poor crystallinity, which was easily resorbed *in vivo*. As demonstrated by TEM [Figure 3E(a)], the prepared mineralized collagen fibril existed as the coarse bundles, which was highly similar to those in the woven bone. Meanwhile, the typical face-on and edge-on image of mineralized collagen fibril could be detected. The periodical deposition of nHA with 67 nm spacing is shown in Figure 3E(b), and the plate-shaped nHA distributed along the collagen fibril [Figure 3E(c)]. In the SAED pattern (Supplementary Figure S2C), the ring-shaped diffraction was associated with (211) of nHA. The bright diffraction points were ascribed to (002) and (004), respectively. The *c*-axis of crystals, aligning from above-left to bottom-right, was the preferential orientation and parallel to the longitudinal direction of the collagen fibril. To further analyze the crystal at plane level, HRTEM observation was conducted. The HRTEM image (Supplementary Figure S2D) showed that plenty of parallel lattice planes were vertically oriented to the longitude of the collagen fibril. The lattice place was 0.34 nm corresponding to the (002), confirming that *c*-axis of nHA oriented parallel to the longitude of the collagen fibril. TGA result presented that approximately 71% inorganics was remained (Figure 3F). The prepared antigen-extracted mineralized collagen fibril was free of cytotoxicity (Figure 3G).

### The Construction of IWBLH

**FIGURE 4:**
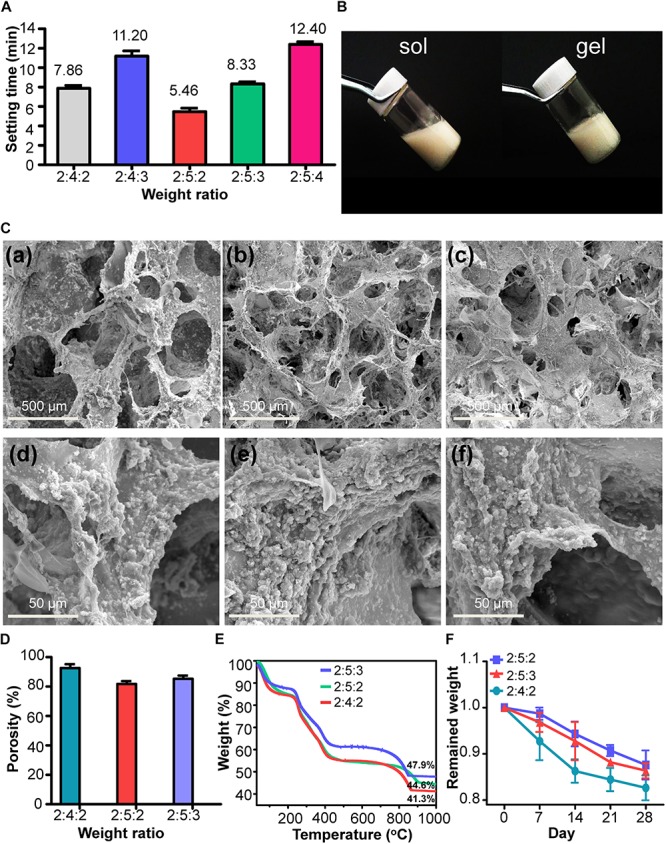
The physiochemical characterization of IWBLHs. **(A)** The sol–gel transition times of various IWBLHs, the weight ratio indicated SA: ACP: mineralized collagen fibril. **(B)** The gross images of IWBLHs before and after gelation. **(C)** Representative SEM images of IWBLHs. **(a,d)**, **(b,e)**, and **(c,f)** represented IWBLHs with weight ratio of SA to ACP to mineralized collagen fibril was 2:4:2, 2:5:2, and 2:5:3, respectively. **(D)** Porosity and **(E)** TGA results of IWBLHs, the weight ratio indicated SA: ACP: mineralized collagen fibril. **(F)** *In vitro* degradation test of IWBLHs (*n* = 3, experiments performed in triplicate. The bars show the mean ± SD).

The sol–gel transition times of IWBLHs with various weight ratio of component are shown in Figure 4A and Supplementary Figure S3. Compared with the gelation time of SA/ACP hydrogel, the addition of mineralized collagen fibrils prolonged the transition times. With the increase of weight ratio of mineralized collagen fibrils, the gelation time extended. The elastic modulus of IWBLHs could reach around 100 Pa. Based on the recommended setting time range, three groups of IWBLHs were selected and subjected to further characterization. The IWBLH was featured by an excellent fluidity in sol state, and maintained the integrity of shape after setting (Figure 4B). Compared with the microstructure of SA/ACP hydrogel, a denser scaffold was obtained after adding mineralized collagen fibrils (Figure 4C). The macropore size was not significantly altered. The surface roughness of pore wall remarkably enhanced. The porosity of three types of IWBLHs were all above 80%, revealing the favorable interconnection of scaffolds (Figure 4D). Approximately 41–48 wt% of mineral was contained in IWBLHs (Figure 4E). The *in vitro* degradation test showed that up to 4 weeks, only less than 20% mass of IWBLH was lost (Figure 4F).

### *In vitro* Biological Activity Test of IWBLHs

**FIGURE 5:**
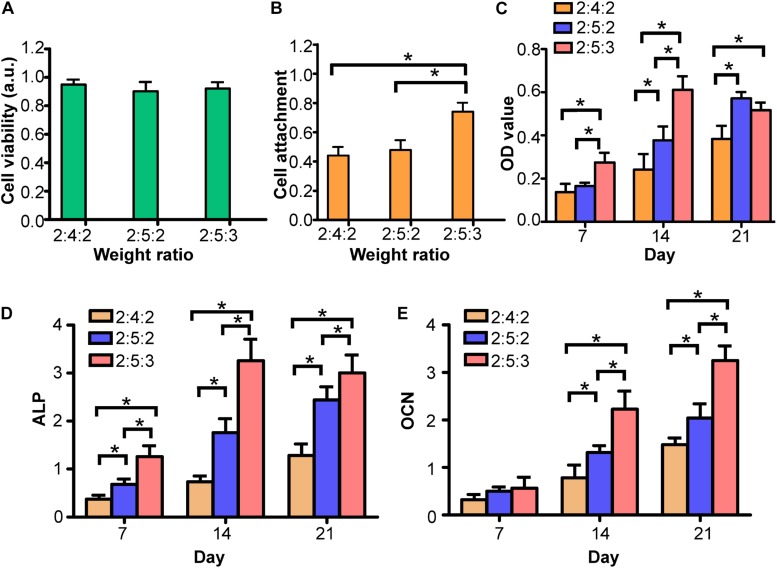
*In vitro* biological activity test of IWBLHs. **(A)** The cell toxicity result of extract of various IWBLHs. **(B)** The BMSCs adhesion results of IWBLHs. **(C)** BMSCs proliferation result of IWBLHs. **(D)** ALP and **(E)** OCN of BMSCs growing on various IWBLHs, the weight ratio indicated SA: ACP: mineralized collagen fibril (*n* = 3, experiments performed in triplicate. The bars show the mean ± SD, *p < 0.05).

The extract of three types of IWBLHs exhibited no cytotoxicity on MG63 cell (Figure 5A), suggesting the good biocompatibility. Compared with the other two IWBLHs, the IWBLH with the highest content of mineralized collagen fibril (the weight ratio of SA: ACP: mineralized collagen fibril was 2:5:3) significantly promoted the BMSCs adhesion (Figure 5B) and proliferation at 7 and 14 days (Figure 5C). At 21 days, the BMSCs proliferation in IWBLH with the highest content of mineralized collagen fibril started to descend, which may be related with the enhanced osteogenic differentiation. Compared with other two IWBLHs, the IWBLH with the highest content of mineralized collagen fibril could effectively induce the expression of early stage (ALP) and late stage (OCN) of osteoblast differentiation marker (Figures 5D,E). Based on the physiochemical and biological test result above, the IWBLH with the highest content of mineralized collagen fibril was selected to conduct the subsequent *in vivo* test.

### *In vivo* Assay of IWBLHs

**FIGURE 6:**
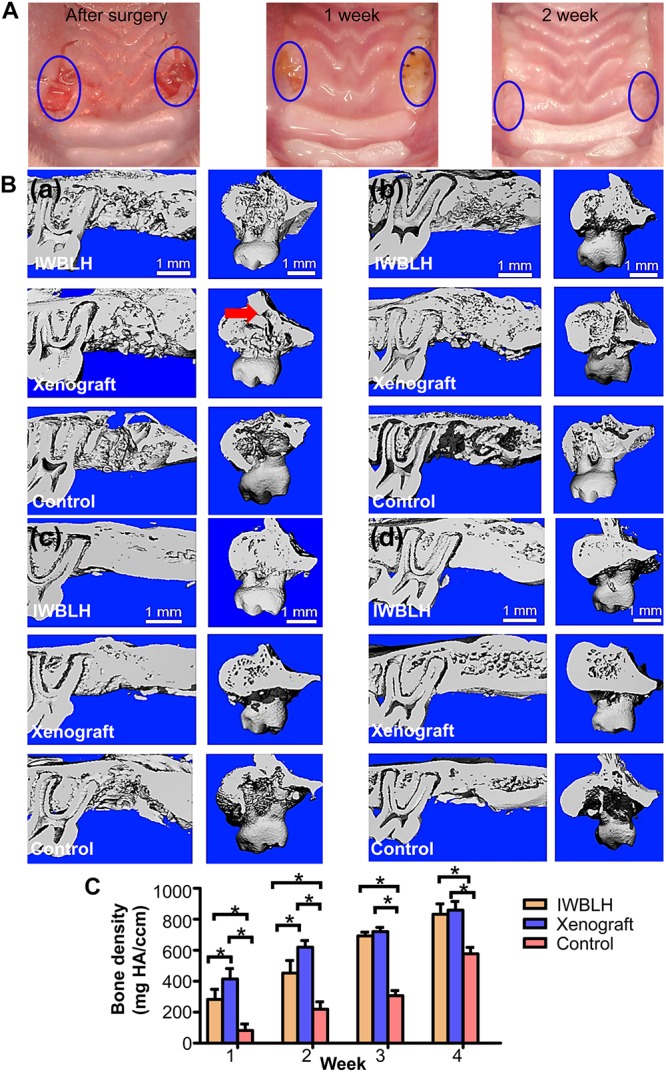
**(A)** The gross observation of tooth socket healing the after surgery. The left tooth socket was grafted with IWBLH, no bone substitute was grafted in the right socket. **(B)** The micro CT images of tooth socket after first week **(a)**, second week **(b)**, third week **(c)**, and fourth week **(d)**. The red arrow indicated the cavity that was not filled by the xenograft. **(C)** The bone density of tooth socket (*n* = 6. The bars show the mean ± SD, *p < 0.05).

Compared with the control group, no obvious redness and swelling phenomena as well as the rejection of material were observed in the tooth socket grafted with IWBLH (Supplementary Figure S4 and Figure 6A), and the oral mucosa was completely healing after 2 weeks, indicating that IWBLH was free of immunogenicity. Evaluated by micro CT (Figures 6B,C), with the prolongation of healing time, the bone density of tooth socket was gradually increased. At week 1 post-implantation, contrary to the empty socket of control group, the sockets of IWBLH and xenograft group were filled with bone substitute. Although the socket density of xenograft group was significantly higher than that of IWBLH group, some apical region of tooth socket was not fully filled by xenograft (pointed by the red arrow in Figure 6B). At second week, the bone density of xenograft group was still higher than other two groups. However, at third week, no significant difference of socket bone density was found between IWBLH and xenograft group. The tooth socket of IWBLH group was almost healed. At fourth week, the tooth socket of IWBLH and xenograft group was completely healed and the socket density continued to increase, whereas only apical half of socket in the control group was occupied by the bone.

**FIGURE 7:**
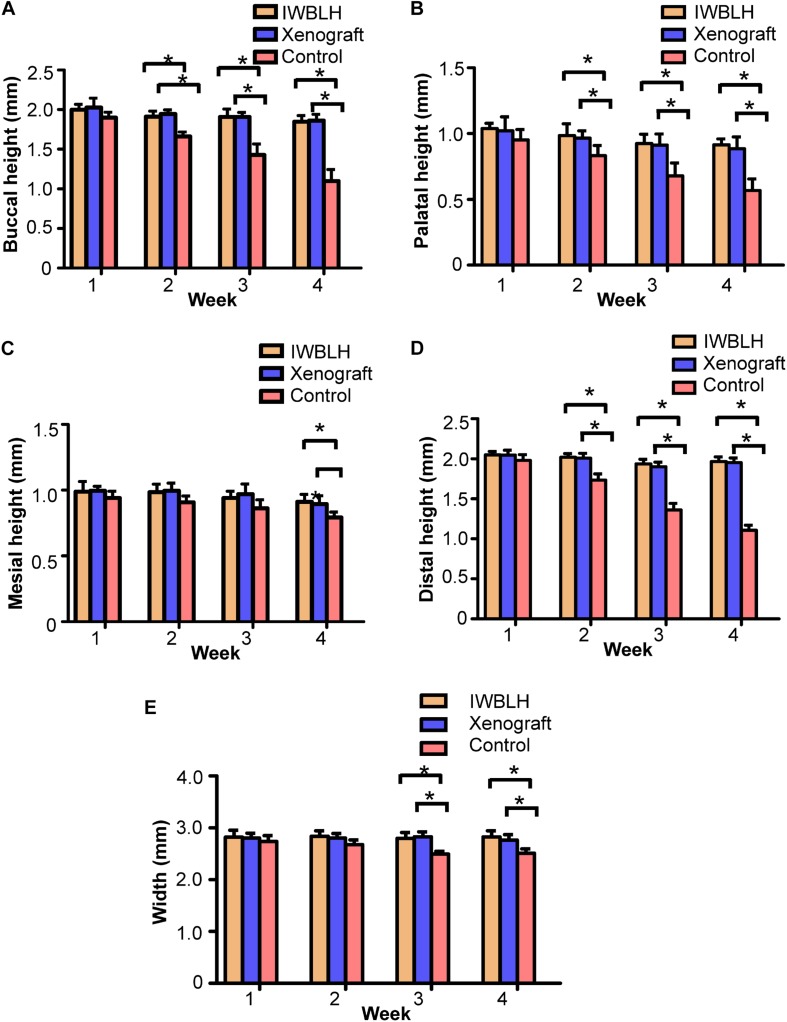
The measurements of **(A)** buccal, **(B)** palatal, **(C)** mesial, and **(D)** distal height as well as **(E)** the width of residual alveolar ridge (*n* = 6. The bars show the mean ± SD, *p < 0.05).

Meanwhile, the function of reducing the residual alveolar ridge resorption of IWBLH was also analyzed *via* micro CT. Shown in Figure 7, there was no statistical difference of height and width among three groups at first week after surgery. However, compared with the control group, the resorption of buccal, palatal, and distal height of alveolar bone in both IWBLH and xenograft group were significantly decreased at second week after surgery. The width and mesial height of alveolar bone started to exhibit a significant difference in IWBLH and xenograft group at third and fourth week, respectively. Notably, IWBLH exhibited a comparable ARP effect with xenograft during the whole healing process.

**FIGURE 8:**
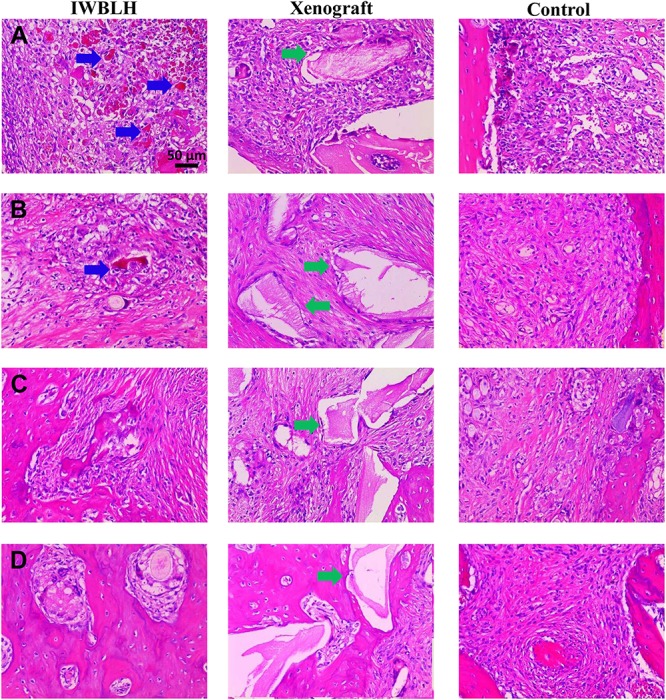
The HE staining of tooth socket of IWBLH, xenograft and control group at **(A)** first week, **(B)** second week, **(C)** third week, and **(D)** fourth week after surgery. The blue arrow indicates the remained IWBLH in the tooth socket, and the green arrow refers to the residual xenograft.

The bone formation and remodeling property of IWBLH were further investigated by HE staining (Figure 8). At first week after surgery, a considerable amount of IWBLH and xenograft were detected in tooth socket, which were covered by granulation tissue. Start from the second week, the granulation tissue was gradually replaced by connective tissue. Of note, only a few IWBLH was observed in socket at week 2 post-implantation, which was completely disappeared at the third week, and the tooth socket was almost filled with bone at fourth week. At week 4 post-implantation, only limited bone was formed in socket in control group. There was still plenty of blocks which could not be remodeled in the xenograft group. Additionally, the result of Masson trichrome staining was consistent with that of the HE staining at week 4 post-implantation (Supplementary Figure S5). Numerous new bone tissue and even some mature bone could be observed in IWBLH and xenograft group. On the contrary, more connective tissue was found in control group instead of bone.

## Discussion

The bone substitute is of essence in ARP technique. Although the capacity to promote the osteogenesis is the prerequisite of bone substitute to accelerate the socket healing thereby preventing the resorption of alveolar bone, the remodeling performance is now gaining increasing interest (Zhang L. et al., 2019). Grafting the substitute with limited or no remodeling property occupies the space of bone, thus potentially interfering with the vital bone formation and severely delaying the subsequent implantation. As documented by previous report, despite alveolar bone was somewhat preserved, only 12% volume of tooth socket was occupied by new bone at 3 months after grafting xenograft, which was significantly lower than 50–60% new bone occupation in the untreated socket (Chappuis et al., 2017). Some studies even observed that the bone substitute remained inside the socket up to 44 months, which may jeopardize the initial stability of dental implant (Zhou et al., 2017). Unfortunately, most bone substitutes employed in ARP were featured by the limited remodeling property. Therefore, constructing the bone substitute with outstanding osteogenic capacity and suitable remodeling property is urgently needed. Woven bone is a transitional bone during the socket healing. The component (ACP, nHA, and collagen protein) and the hierarchical structure (porous scaffold and randomly distributed mineralized collagen fibril) could provide a favorite microenvironment for bone formation. More importantly, the woven bone is rich in ACP characterized by a higher solubility. The nHA of mineralized collagen fibril is featured by the poor crystallinity. The mineral content of woven bone is low and the organization of woven bone is disordered. All these factors make this immature bone easily remodeled within 3–6 months. Therefore, mimicking woven bone was supposed to be a feasible strategy to endow the substitute with excellent ARP effect and suitable remodeling property.

The component and hierarchical structure are the key issues need to be simulated when constructing the biomimetic material (Wegst et al., 2015; Wingender et al., 2016; Ling et al., 2018; Zheng et al., 2018). ACP was a critical component and abundant in woven bone besides collagen and nHA. In our study, as investigated by XRD, FTIR, SEM, and TEM (Supplementary Figure S1), ACP was successfully constructed. Equally importantly, the resulting ACP could transform into nHA in PBS within 24 h. Since the phase transformation involved the dissolution–reprecipitation process and surface-mediated transformation mechanism (Combes and Rey, 2010), calcium and phosphate could release from the ACP during transformation process, which favored the SA hydrogel formation, exerted the bioactivity during osteogenesis, and precipitated in the surface of the hard tissue mediating the bonding between substitute and bone (Supplementary Figure S1D).

As the fundamental composition of bone, collagen and nHA assembled into the mineralized collagen fibril acting as the building block (Zhang Y. et al., 2018). Currently, mineralized collagen fibril was fabricated *via* the combination of self-assembly of collagen and polymer-induced liquid-precursor (PILP). Although this bottom-up method did tremendously deepen the understanding of mechanism of bone formation and greatly promote the development of mimicking bone ECM (Niu et al., 2017), it still hindered by several obstacles when constructing the biomimetic woven bone. During the fabrication, the concentration and pH of PILP should be precisely controlled (Liu et al., 2016b), which may be unfit for the large-scale manufacturing. The fabricated mineralized collagen fibril lacks of non-collagen proteins (N) which plays the pivotal role during osteogenesis. The fibrils generated through self-assembly tend to exist as the individuals rather than the coarse bundle of fibrils presenting in woven bone (Reznikov et al., 2014). To address these drawbacks, the top-down approach was employed in this study. Through this approach, up to kilogram of mineralized collagen fibril could be generated every time. Although a portion of the protein was removed during the antigen extraction process, theoretically, some NCPs may still remain and continue to exert the bioactivity. As shown in Figure 3 and Supplementary Figure S2, the product possessed of the typical characteristic of both intrafibrillar and extrafibrillar mineralization. The *c*-axis alignment of nHA was parallel to the longitudinal direction of the collagen fibril, which was quite similar to the mineralized collagen fibril constructed through bottom-up method and highly alike to that in the natural bone (Zhang et al., 2003; Zhai and Cui, 2006; Qi et al., 2018; Wang Y. et al., 2018). Meantime, the coarse fibrils could be obtained to highly imitate the woven bone. The inorganic mineral in bone tissue is mainly made up of poorly crystalline hydroxyapatite (Sprio et al., 2016). The poor crystallinity can increase the solubility of hydroxyapatite at the physiological pH and is easily absorbed under the activity of osteoclast. Thus, it has been demonstrated that the formation rate of new bone is somewhat in balance with the resorption rate of poorly crystalline hydroxyapatite (Zhao et al., 2011). The mineralized collagen fibril was characterized by a poor crystallinity (Figure 3D), indicating that mineralized collagen fibril was easily remodeled *in vivo*. The mainly concerned issue from this approach is the biocompatibility. As demonstrated by SEM and TGA (Figure 3 and Supplementary Figure S2), the antigen of bone, mainly composed of fat and protein, been effectively removed, without impairing the physiochemical property of mineralized collagen fibril. Besides, the gross monitoring of socket healing verified that the mineralized collagen fibril constructed *via* top-down approach was deprived of antigen and could be safely employed.

Another pivotal affair in mimicking the hierarchical structure of woven bone was to construct the 3D ECM architecture (Zhang et al., 2019c). The ECM has a prominent interpore connectivity and an ideal bone substitute should provide a similar interconnected pore structure to support cell attachment and migration as well as the circulation of nutrients (Zadpoor, 2015; Zhang Y. et al., 2019; Zhu et al., 2019). The optimal pore size recommended is recommended over 300 μm and the porosity is preferably in the range from 70 to 90% (Turco et al., 2009; Tian et al., 2019). In our study, the alginate hydrogel was selected to achieve scaffold. The IWBLHs exhibited the anastomosing reticular structure with an average pore size of 300–600 μm and over 80% porosity (Figure 4). The mineralized collagen fibril and ACP were widely and uniformly distributed in the pore wall, which highly mimicked the microstructure of woven bone. The mineral content of IWBLHs was highly closely to that in woven bone (Garcia-Rodriguez and Martinez-Reina, 2017). Due to the complex interaction among mineralized collagen fibril, ACP, and SA, the IWBLHs were structurally stable and weight loss were less than 20% over 4 weeks in PBS, indicating that IWBLHs could provide an integrated architecture for cell. Although numerous hydrogels have been employed in bone regeneration, the majority of them are only designed to act as the scaffold carrying and delivering growth factor to promote osteogenesis (Chen et al., 2019; Pan et al., 2019; Papageorgiou et al., 2019) rather than mimicking the complex bone microenvironment. In our study, an alginate-based hydrogel was employed with the incorporation of ACP and mineralized collagen fibril. As demonstrated by the above results, the component and hierarchical structure of woven bone could be highly mimicked.

Apart from the osteogenic and remodeling property, the clinical manageability is also the crucial aspect designing the ARP bone substitute. With the calcium sustained release from ACP, the alginate could be injected into the irregular tooth socket, filled in socket, and gelled *in situ*. Although the mineralized collagen fibril has somewhat delayed the gelation time *via* competitive binding with calcium ions, a series of desirable gelation time could still be obtained (Figure 4A). Contrary to the granular xenograft that is not easily to manipulate and completely contact the socket (Figure 6), the IWBLH, with the satisfied fluidity, could maximumly contact with the tooth socket thereby exerting a better osteoconduction.

Following the synthesis and the physiochemical tests screening, three types of IWBLHs were selected and subjected to further *in vitro* biological evaluation. Osteo-progenitor cells exert a vital role in bone remodeling (Han et al., 2019). During the healing process, osteo-progenitor cells are recruited and fairly abundant in woven bone. With the osteogenic activity of osteoprogenitor cells, the collagen protein and calcium phosphate are produced, and these basic bone compositions assemble to the complex hierarchy and eventually replace the woven bone, leading to the bone remodeling (Schindeler et al., 2008; Cui et al., 2020). Therefore, the effect of IWBLH on activity of osteo-progenitor cells is pivotal. The cell anchorage was the critical step orchestrating the subsequent proliferation and bone formation. Due to the minimal protein adsorption capacity of alginate (Rowley et al., 1999), cells were unable to adhere to the pure alginate hydrogel. Therefore, the cell attachment property was endowed by ACP and mineralized collagen fibril. Compared with ACP, increasing the weight ratio of mineralized collagen fibril remarkably enhanced the cell adhesion (Figure 5). Although ACP has been reported to possess an increased osteoblast adhesion than HA (Balasundaram et al., 2006), the mineralized collagen fibril, with complicated organization of collagen and nHA, exhibited a superior performance. The collagen was rich in RGD peptides which mediated the interacting between integrin of cell and material (Li et al., 2017). nHA could not only directly promote cell adhesion but also passively adsorb RGD peptide. For the differentiation, the increased weight ratio of ACP and mineralized collagen fibril significantly promoted the osteogenic differentiation of BMSCs. The calcium release from the ACP could stimulate osteoblastic differentiation and the hierarchical nanostructure of mineralized collagen fibril could also induce the commitment toward the osteoblast phenotype (Liu et al., 2016a). Based on these results, the IWBLH with the most outstanding biological performance was selected and further investigated in the *in vivo* study.

Since the xenograft was made up of radiopaque inorganic minerals, the bone density of socket investigated by micro CT was significantly higher in xenograft group than IWBLH during the first 2-week post-grafting (Figure 6). However, no statistical difference was found between xenograft and IWBLH group at third week, indicating that IWBLH did vastly induce the bone formation in socket. The HE staining also confirmed this phenomenon. As for the function to prevent the alveolar bone resorption, IWBLH displayed a potent ARP capacity, which was comparable to the widely used product in market (Figure 7). More importantly, contrary to the xenograft that remained inside the socket even after 4 weeks, the IWBLH could be gradually remodeled and completely replaced by the vital bone (Figure 8 and Supplementary Figure S5). The above results proved that mimicking the woven bone was a feasible strategy to achieve ARP effect with desirable remodeling performance. This IWBLH stands as a promising bone substitute for ARP application. The limitation of current IWBLH was that its mechanical strength was relatively weak. Thus, it could be only used in the non-loading bearing area such as tooth socket. In future, more effort will be devoted to enhance its strength to extend its application in dentistry.

## Conclusion

To improve the osteogenesis and address the limited remodeled property of current bone substitute employed ARP technique, the biomimetic woven bone substitute was synthesized in this study. Meanwhile, to further facilitate the clinical manageability, the injectable hydrogel was introduced and an injectable biomimetic woven bone was constructed. Using ACP, mineralized collagen fibril and SA as the ingredient, the component and hierarchical structure of woven bone were highly simulated. The resulting IWBLH exhibited a comparable ARP effect with xenograft that was widely used and possessed a suitable remodeling performance *in vivo*. In the future, more effort will be devoted to further increasing the mechanical strength of IWBLH, in order to expand its application in loading bear bone defect area.

## Data Availability Statement

The datasets generated for this study are available by requesting to the corresponding author.

## Ethics Statement

The animal study was reviewed and approved by the Animal Research Committee of the West China School of Stomatology, Sichuan University.

## Author Contributions

WT, ZZ, and YL contributed substantially to the conception and design of the experiments. TY, PX, and ZW conducted all experiments and wrote the manuscript. ZH and YW conducted data analyses. WC revised and modified the draft.

## Conflict of Interest

The authors declare that the research was conducted in the absence of any commercial or financial relationships that could be construed as a potential conflict of interest.
