# Supplementary material for: The Injectable Woven Bone-Like Hydrogel to Perform Alveolar Ridge Preservation With Adapted Remodeling Performance After Tooth Extraction
Source: Front Bioeng Biotechnol. 2020 Feb 21;8:119. doi: 10.3389/fbioe.2020.00119 (PMC7047753; doi:10.3389/fbioe.2020.00119)
Supplement: Supplementary file 1 [file Table_1.DOCX]

Supplementary Material

**Supplementary Figure 1.** The characterization of ACP. (A) XRD and FTIR spectra, (B) SEM, (C) TEM, the insert was SAED. In (A)-(C), (a) indicated the fresh synthesized ACP, (b) represented the ACP conversed in PBS after 24 h. (D) SEM image of bone immersed in ACP dispersion. The insert was EDS result.

**Supplementary Figure 2.** (A) TGA and (B) XRD of bone during antigen extraction process**.** The SEM image of bone during the process. (a) fresh bone, (b) bone subjected to the first methanol and chloroform process, (c) bone subjected to hydrogen peroxide process and (d) bone subjected to second methanol and chloroform process. (C) The TEM image of mineralized collagen fibril and corresponding SAED pattern. (D) HRTEM image of mineralized collagen fibril. The white arrow indicated the longitude direction of collagen fibril.

 **Supplementary Figure 3.** The curves of storage modulus (G’) and loss modulus (G’’) versus time of IWBLH. The temperature was set as 37 ^o^C. The weight ratio of SA: ACP: mineralized collagen fibril in A-E was 2:4:2, 2:4:3, 2:5:2, 2:5:3 and 2:5:4, respectively.

**Supplementary Figure 4.** The surgery procedure of creating the standard rat model of tooth socket and grafting IWBLH. (A) After the anesthesia, the rat mouth was opened via a homemade mouth gag. (B) the gingiva was separated from tooth using the explorer, followed by luxating M1. (C) Using the hemostatic forceps, M1 from both sides were extracted in a minimally invasive approach. (D) The M1 was completely extraction with no root fracture. (E) The interradicular septum of M1 was removed via a #4 round dental bur until the depth from cementoenamel junction of M2 to socket bottom reached 2 mm. (F) A standard model of tooth socket was established. (G) IWBLH was injected into the left M1 tooth socket, and the right socket was set as the blank control. (H) After covering the collagen membrane, the gingiva was sutured using 5-zero polyglactin interrupted stitches. (I) The image of mucosa after surgery.

S**upplementary Figure 5.** The Masson trichrome staining of tooth socket of IWBLH, xenograft and control group at fourth week after surgery.
